# Supplementary material for: Fano Resonance-Assisted All-Dielectric Array for Enhanced Near-Field Optical Trapping of Nanoparticles
Source: ACS Photonics. 2023 Nov 14;10(12):4322–8. doi: 10.1021/acsphotonics.3c01126 (PMC10740001; doi:10.1021/acsphotonics.3c01126)
Supplement: Supplementary file 1 — ph3c01126_si_001.pdf [file ph3c01126_si_001.pdf]

Supplementary Information: Fano resonance-assisted all-dielectric array for enhanced near-field optical trapping of nanoparticles

Donato Conteduca,[†,‖] Saba N. Khan,[‡,‖] Manuel A. Martínez Ruiz,[‡] Graham D. Bruce,[‡] Thomas F. Krauss,[*,†] and Kishan Dholakia[*,‡,¶,§]

†*School of Physics, Engineering and Technology, University of York, Heslington, YO10 5DD, York, UK*

‡*SUPA, School of Physics and Astronomy, University of St Andrews, North Haugh, St Andrews KY16 9SS, UK*

¶*School of Biological Sciences, University of Adelaide, Adelaide, South Australia, 5005, Australia*

§*Centre of Light for Life, University of Adelaide, 5005, Australia*

‖*These authors contributed equally to this work.*

E-mail: thomas.krauss@york.ac.uk; kd1@st-andrews.ac.uk

This supplementary document provides comprehensive details regarding the simulation conducted to analyze the optical response of the quadrupole array, the fabrication process of the nanostructure device, and the experimental setup employed to achieve near-field optical trapping with the assistance of Fano resonance effect.

S1: Optical response of the quadrupole array

The optical properties of the array were calculated for different asymmetry values of the unit cell. The quadrupole configuration was realized by mirroring a pair of elliptical meta-atoms along the vertical direction. If the angular orientation of each meta-atom is null ($\theta = 0°$), a bound state in the continuum (BIC) mode is supported, which is symmetry protected, a condition that is usually associated with an infinite Q-factor and a non-radiated state. When $\theta \neq 0$, the structure is asymmetric and a quasi-BIC mode is supported[1] and the value of the Q-factor and radiation is proportional to the asymmetry of the unit cell.

In our study, the introduction of a mirrored pair of elliptical meta-atoms allows the preservation of the typical behavior of a quasi-BIC mode, together with the possibility of controlling the energy confinement in the central gap of the quadrupole, as previously verified with Huygens meta-surfaces with a similar configuration.[2]

The largest change in the optical properties of the array is obtained by tuning the angular orientation of the meta-atoms. As anticipated, the simulation revealed an exponential decay of the Q-factor and an increase in the resonance amplitude as θ was increased (refer to Fig. S1(a) and (b)). To improve the trapping efficiency of the quadrupole structure, it is crucial to maximize both the resonance amplitude and the Q-factor. However, a very high Q-factor values can be disadvantageous as they may facilitate the escape of the nanoparticle from the trapping site.

The optimized design here provides a Q-factor > 100 and a resonance amplitude > 0.8 (Fig. S1(b)). The energy confinement shown in Fig. S1(c) and (d) confirms the strong interplay of Q-factor and resonance amplitude on the optical gradient in the trapping site. The condition of low asymmetry ($\theta = 10°$) provides a high Q-factor but at the expense of limited resonance amplitude, which corresponds to a decrease of the field by a factor 1.4 compared to the condition $\theta = 20°$. In contrast, a larger asymmetry ($\theta = 30°$) results in a higher resonance amplitude. However, there is no improvement in the gradient field due to a decrease in the Q-factor. Thus, we have selected $\theta = 23°$ as the optimal compromise,

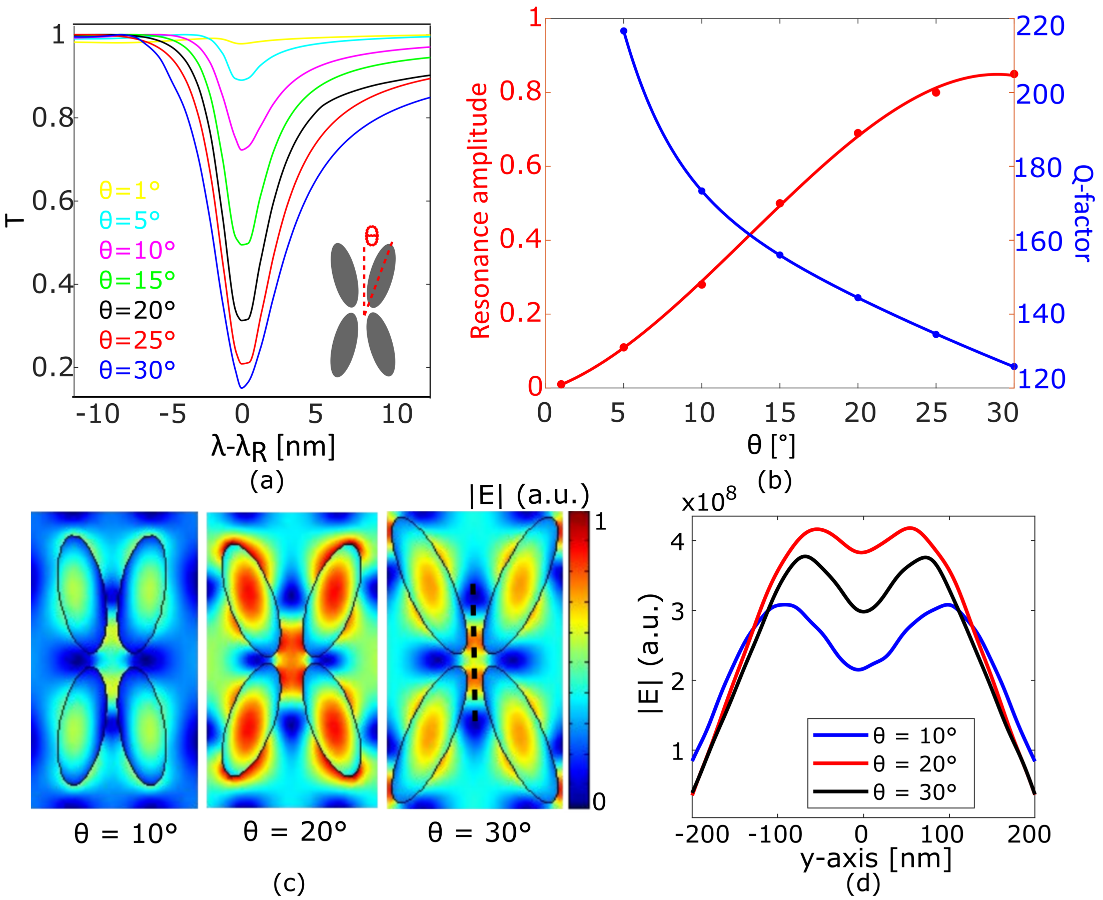

Figure S1: (a) Transmission spectra of a quadrupole array with a different angle orientation θ of the meta-atoms. (b) Q-factor and resonance amplitude as a function of θ. (c) Energy confinement in the unit cell for $\theta = 10°$, $20°$ and $30°$, calculated in the xy plane in the middle of the quadrupole structure and (d) correspondent field distribution in the trapping site, calculated in the dotted line in (c).

balancing the highest gradient field exerted in the central gap with minimal interaction between adjacent unit cells. By making this selection, the formation of multiple trapping sites across the surface of the quadrupole meta-atoms is prevented, while simultaneously preserving a greater trapping efficiency.

S2: Polarization dependence of the quadrupole array

The modes of the quadrupole array exhibit polarization dependence, as demonstrated in Fig. S2. As the light field's polarization state is varied from horizontal to vertical, the efficiency

of the Fano-resonant dip in transmission diminishes. This capability of the quadrupole array can be used to dynamically control the field localization and thereby the trapping efficiency.

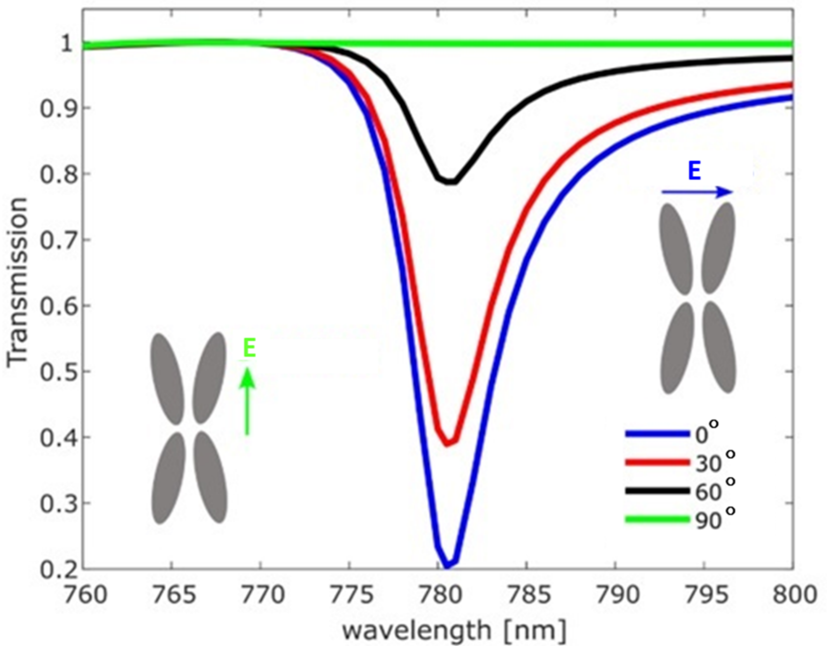

Figure S2: Simulated transmission spectra of the quadrupole array for different polarization states of the excitation beam.

S3: Thermal Distribution in quadrupole Array

We utilized a Multiphysics approach with 3D finite element method simulations to estimate the temperature-increase distribution surrounding the quadrupole (Si) nanostructure. Firstly, the simulation of a quadrupole array with multiple elements is carried out and the electromagnetic confinement obtained at the resonance is used as the initial solution for the thermal analysis. For the thermal study, the computational volume is around 100 times larger than that assumed in the optical study. The distribution in the cross-section through each nanostructure center and the section of the entire array is presented in Fig. S3 at an intensity that is typical in experiments (I= 5 mW/μm^2). According to the simulation, the

maximum temperature increase is estimated to be around 2.35 K, with the structure exhibiting a maximum gradient of 0.9 K. Notably, this temperature rise is nearly 2 orders lower in magnitude than the plasmonic structures not having a heat dissipation sink attached to the structure.[3–5] The low heating in all-dielectric structures such as the one presented here will allow higher laser intensities to be used for biomedical applications or for exploring nonlinear processes in trapped nanoparticles. On the other hand, the Joule heating in our quadrupole array is found to be slightly more than that of the all-silicon structure.[3] This is due to the following reasons (1) the Si nanoantenna was on a Si substrate with a much greater thermal conductivity than that of the quadrupole nanostructure which was on borosilicate glass. (2) The quadrupole array was composed of hydrogenated amorphous silicon whose optical properties are different from the crystalline Si considered for Si nanoantennas.

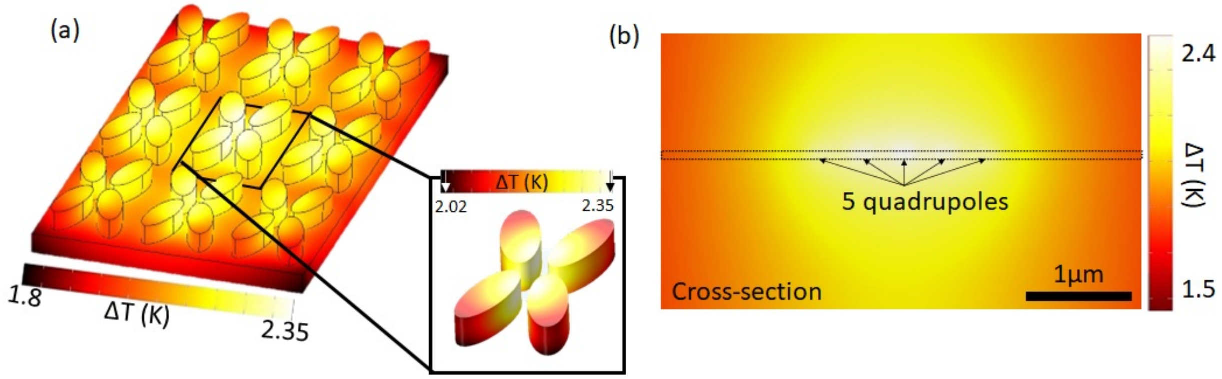

Figure S3: Simulated thermal map of the 5 × 5 quadrupole array at a power density of 5 mW/μm^2, which is a typical value in our experiments. (a) The thermal distribution shown here is for a 3x3 array, and a zoomed image revealed the highest gradient occurring around the central core of the array. (b) Cross-sectional view of five quadrupole structures.

S4: Comparison between the quadrupole unit cell and the array

A comparison was made between the energy confinement of the quadrupole structure in an array configuration and that of a single element (unit cell). The numerical results vali-

date that the array configuration yields a ten-fold enhancement of the electric field (E-field) compared to the single element. This enhancement is attributed to the array's robust resonant behavior (see Fig. S4). Furthermore, the light confinement in the single quadrupole is also largely distributed around the elliptical meta-atoms instead of the central gap, which corresponds to a weaker optical gradient in the trapping site.

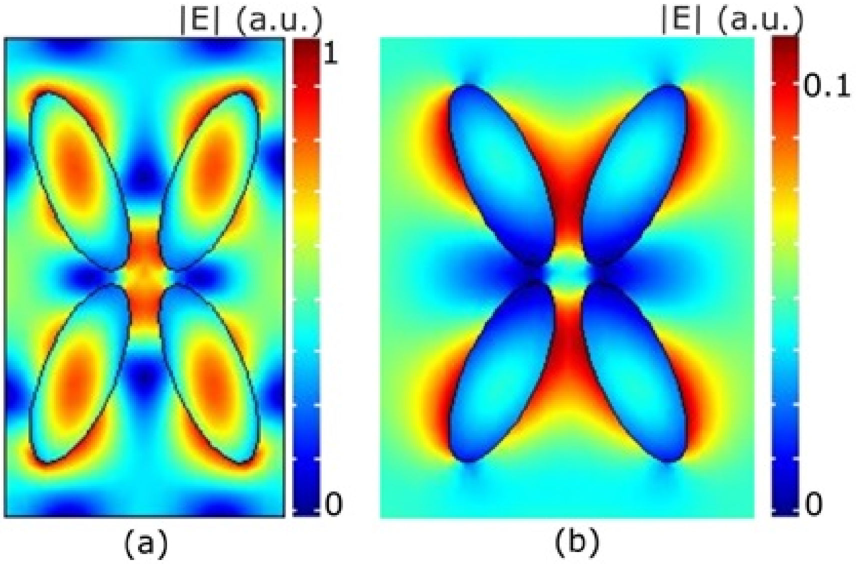

Figure S4: Field confinement in (a) an infinite array of quadrupole structures and (b) in a decoupled single quadrupole.

S5: Trapping stability

As shown in Fig. S5(a) and (d), the confined field has a pronounced double-lobed structure along the y-axis, which is designed to hold the particle as far from the meta-atoms as possible. For small particles, the local minimum will result in an anti-trap and the particle will be pulled into one lobe or the other (upper panel of Fig. S5(b) and (c)). However, for larger particles, such as the 100nm-diameter particles this structure was designed for, the local minimum is effectively averaged out (lower panel of Fig. S5(b) and (c)), allowing the particle to sit stably between the two field maxima. Along the z-axis, the particle experiences a near-harmonic potential (Fig. S5(e) and (f)) which draws the particle to an equilibrium

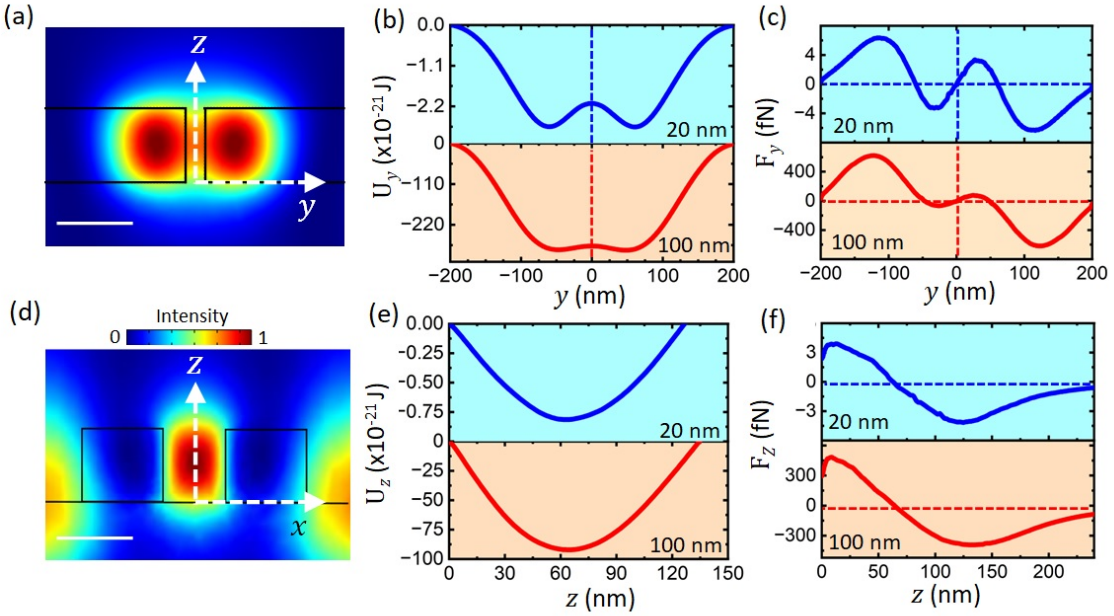

Figure S5: (a) and (d) represent the distributions of near field intensity of a Fano resonant quadrupole array in the yz and xz planes respectively. The scalebar is 120 nm. Panel (b) and (d) are the curves for trapping energies as a function of position along the y and z axes for 20 nm polystyrene particles (cyan background) and a 100 nm (orange background). Panels (c) and (f) show the corresponding force curves for 20 nm and 100 nm nanoparticles as a function of position along the y and z axes.

height mid-way between the base and the top of the meta-unit (see Fig. S5(d)), and due to the single-lobed structure there is no phenomenological difference in the vertical trapping forces on particles of different sizes.

S6: Simulated performance of quadrupole array for smaller nanoparticles

The simulations are performed to evaluate the performance of the quadrupole array with smaller nanoparticles. The results are summarized in Table 1, with the first three columns illustrating different scenarios while maintaining the proposed dimensions of the quadrupole nanostructure. As expected, the trap stiffness decreases for smaller particles. Furthermore,

the last two columns depict that bringing the meta-atoms closer together results in a more tightly confined light field, enabling the creation of stronger traps for smaller particles. Notably, the geometry featuring the overlapping vertical pair of elliptical poles (see last column in Table 1) represents the optimized design for trapping 20 nm nanoparticles. It is noteworthy that the trap stiffness for this specific geometry, targeting 20 nm nanoparticles, is of a similar magnitude to that demonstrated for 100 nm particles with the optimum geometry. In all these cases, the estimated trap stiffness values are obtained considering a power density of 1 mW/μm^2.

Table 1: Different quadrupole unit cell configurations and their near-field trapping performance for smaller nanoparticles estimated at a power density of 1mW/μm^2. A common 100 nm scale bar is used for all design configurations.

Quadrupole Design	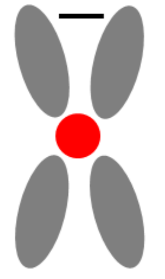	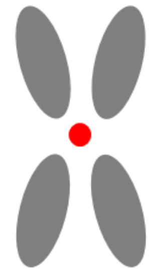	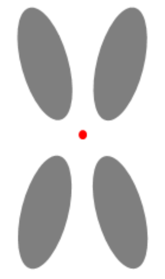	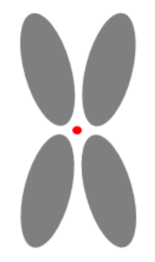	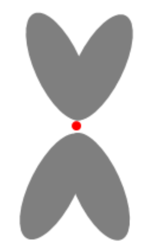
Nanoparticle size	100 nm	50 nm	20 nm	20 nm	20 nm
Trap Stiffness	5.1 fN/nm	0.7 fN/nm	0.05 fN/nm	0.5 fN/nm	2.9 fN/nm

S7: Fabrication of the quadrupole nanostructures

The dielectric quadrupole array was realised in hydrogenated amorphous silicon (a-Si:H) with a thickness of 120 nm on a 500 µm Borofloat glass (BF33) substrate (PI-KEM). The sample is cleaned with a piranha solution (1:3 hydrogen peroxide:sulfuric acid) for 10 minutes and rinsed in deionised water, acetone and isopropanol. The pattern of the nanostructures is defined by e-beam lithography. The positive resist ARP-9 (Allresist GmbH) was spun at 4000 rpm for 60 seconds and subsequently baked at 180°C for 5 minutes to achieve a thickness of 200 nm. A 60 nm layer of AR-PC 5090 (Allresist GmbH) is spin-coated at 2000

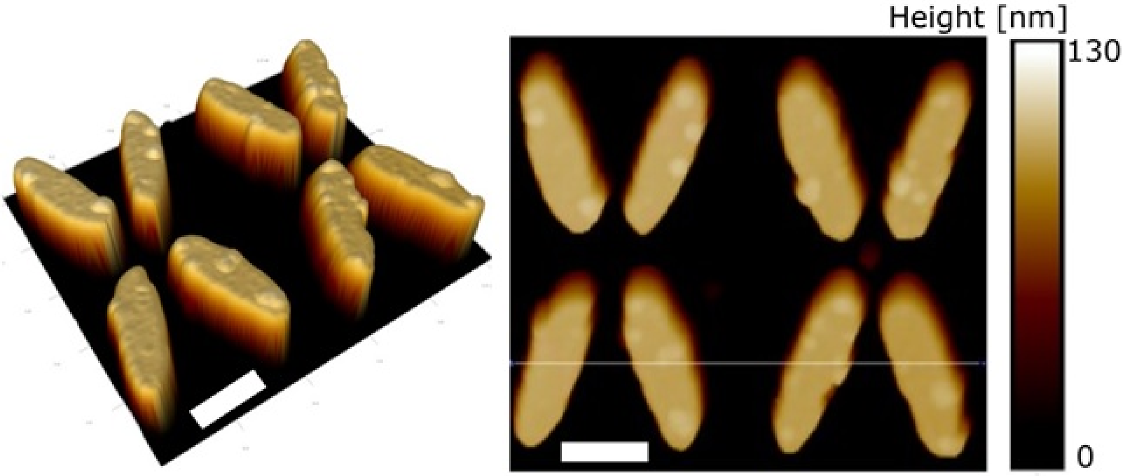

Figure S6: Angled view (left) and top view (right) of two quadrupole structures in a-Si:H. The scalebar is 200 nm.

rpm for 60 seconds and baked at 90°C for 2 minutes for charge dissipation during the e-beam exposure. For the e-beam exposure (50kV Voyager, Raith GmbH) a current of 120 pA and a dose of 130 $\mu C/cm^2$ is used. After washing the sample to remove the charge dissipation layer, we developed it with xylene for 2 minutes and rinse in isopropanol. The quadrupole structure is transferred into the a-Si:H layer by reactive ion etching with a gas mixture of CHF_3:SF_6 = 14.5 sccm: 12.5 sccm for 80 seconds with a voltage of 188V and a chamber pressure of 0.4 mbar. Finally, the remaining resist is removed with 1165 solvent (Microchem) in a sonic bath at 50°C for 10 minutes, then rinse in acetone and isopropanol and a final drying step with nitrogen. An atomic force microscopy (AFM) image of two quadrupole structures is shown in Fig. S6.

S8: Position dependence of Fano resonance characteristics

The Fano resonance characteristics of the quadrupole array were observed to be position dependent in experimental measurements. Figure S7 displays the reflectivity curve of the quadrupole array obtained from three different regions within the array. It is evident that

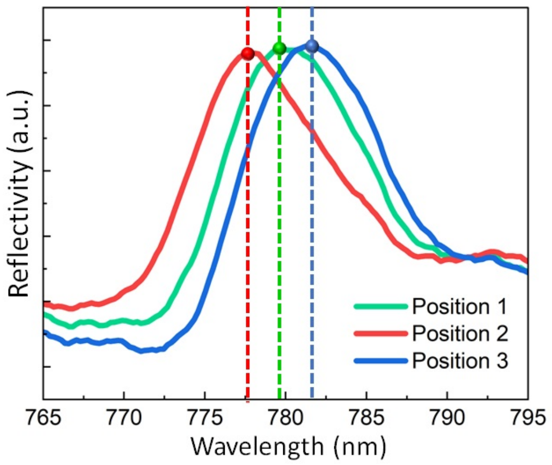

Figure S7: Reflectivity curve showing the position-dependent response of Fano resonance effect.

the peak resonant wavelength shifts from (777.2 ± 0.2) nm to (781.8 ± 0.2) nm. The likely reasons for this position dependence are: (a) fabrication variations, (b) the utilization of weakly focused illumination on the quadrupole array, and (c) the adhesion of nanoparticles to the surface of the quadrupoles. The high sensitivity of the Fano resonance peak to the beam position may be particularly advantageous for future experiments where a tunable laser is not available, and therefore the stiffness can be maximised by judicious choice of illumination location rather than by scanning wavelength.

S9: Transmission characteristics of coupled and decoupled quadrupole array

The experimental comparison of the near-field confinement capability between coupled and decoupled quadrupole arrays is performed by measuring the transmittance through both structures. In Fig. S8, the blue curve represents a quadrupole array where the individual quadrupole unit cells are isolated and do not exhibit significant interaction. The resonant quadrupole array (see red curve in Fig. S8) has a quality factor of 76.

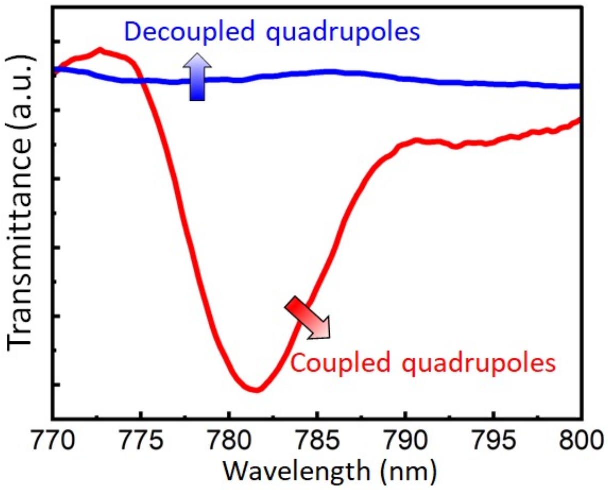

Figure S8: Transmittance characteristics of quadrupole array and decoupled quadrupole structure.

S10: Trapping experiment details

The near field trapping system consist of a tunable, continuous-wave (CW) Ti: sapphire laser weakly focused using a 40X objective lens onto the nanostructure device (quadrupole array), which was mounted on an 3D-translational stage (see Fig. S9). The laser beam is directed to the objective lens using a dichroic mirror (reflects 750 nm and above light only). A half-wave plate (HWP) is used to rotate the polarization of the trapping laser beam. The spot size on the sample plane is approximately 5.1 μm, which illuminates 6x5 unit cells of the nanostructure array. At first, the Fano resonance characteristic of the quadrupole array was probed with the white light source (OSL1 Thorlabs). The transmitted signal through the quadrupole array is collected using a long working distance objective which is then interrogated by a spectrometer. Subsequently, the illumination was switched from white light to the trapping laser using a flip mirror. The trapping laser is tuned close to the resonance wavelength identified with the white light source. Once a particle was trapped, the wavelength of the trapping laser was scanned over the whole range of the Fano resonance curve. The optical trapping process is monitored by observing the nanoparticle fluorescence after a set of filters (dichroic mirrors and short/long/band pass filters) with an electron-

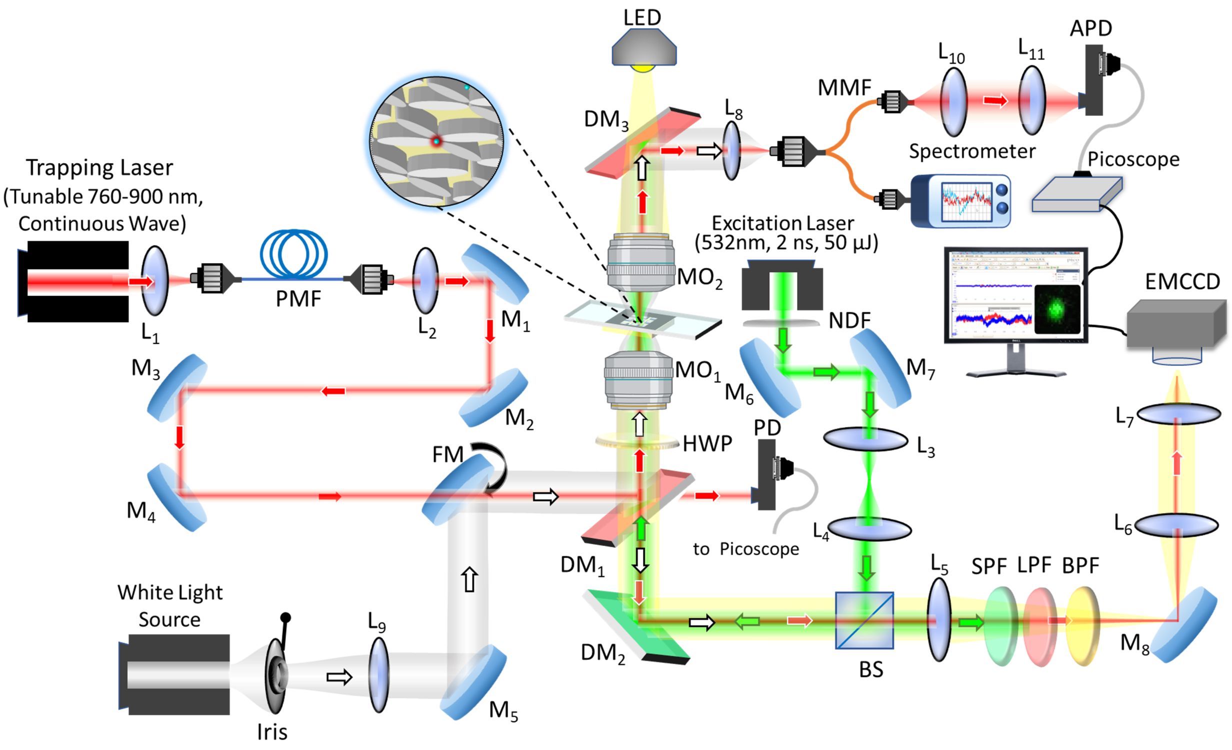

Figure S9: Detailed schematic for the experimental setup used for Fano resonance assisted near field trapping of red-fluorescent (100 nm) polystyrene nanoparticles. Abbreviations are L-lens, M-mirror, MO-microscope objective lens, MMF-multimode fiber, PMF-polarization maintaining fiber, BS-beam splitter, FM- flip mirror, APD-avalanche photodetector, PD- photodetector, SPF-short-pass filter, LPF-long-pass filter, BPF-band-pass filter, NDF-neutral density filter, EMCCD-electron multiplying charge-coupled device, LED-light emitting diode, HWP-halfwave plate.

multiplying camera (EMCCD;iXon Ultra 897, Andor, Oxford Instruments) operated at 30 fps with an exposure time of 30 ms. We controlled both the polarization (using an HWP) and incident intensity (using a filter wheel) of the trapping beam, which was limited to a maximum power density of 15 $mW/\mu m^2$ at the back objective plane. The quick detection of a trapping event was done by collecting the transmitted laser light through a 50$\times$ objective lens (Mitotoyu) and sending it to an avalanche photodiode (APD430A/M, Thorlabs). The APD signal of the reflected laser light was in the opposite direction (step-like jump up or down) to the APD signal of the transmitted laser light (step-like jump down or up). This ensured that the step-like jump was due to an optical trapping event. Next, fluorescence microscopy is performed to track the Brownian movement of the trapped nanoparticle. To do

so, a weakly focused green laser (532 nm wavelength, 2 ns pulse duration, 50 μJ pulse energy) is directed along the same optical path to excite red fluorescent polystyrene nanoparticles (R100, Duke Scientific Corp., 100 nm diameter). The average power at the sample plane due to the green laser is approx 100 μW. The polystyrene nanoparticles are suspended in heavy water with a trace amount of Tween 20 surfactant added and the diluted suspension is ultrasonicated using a bath sonicator. The nanospheres have a coefficient of variation of about 5 percent for 100 nm respectively, according to the manufacturer. The experiment is done with freshly made suspension every time. To prepare the trapping chamber, a vinyl spacer is placed on top of a coverslip. The thickness of the coverslip used is much smaller than the working distance of the illumination objective. Next, 20 μL of the solution of nanoparticles is added to the created "pool". The nanostructure is positioned upside down to close the chamber. The sample was held in place using scotch tape or similar adhesive, to reduce the water currents created when the nanostructure moves. It is recommended to refresh the particle solution every few hours. This practice serves two purposes (a) it helps prevent the sticking of the nanoparticles to the nanostructure, and (b) to replenish the water pool for possible water evaporation during the experiments. Each pixel on the EM-CCD sensor array is 16 μm × 16 μm, which translates to around 0.08 $\mu m \times 0.08\,\mu m$ in the focal plane with the 40× magnification objective and magnification telescopes. The centroid movement of the emitted signal for the trapped nanoparticle is tracked and used to calculate the effective trap stiffnesses along the x and y directions. We observed a distinct step-change in the forward-propagating signal recorded on the Avalanche Photodiode (APD) as a particle entered the trap. For instance, as depicted in Fig. S11, when the trapping laser is close to λ_R in Fig. S10 , we observe a decrease in transmission with the trapping of each additional particle.

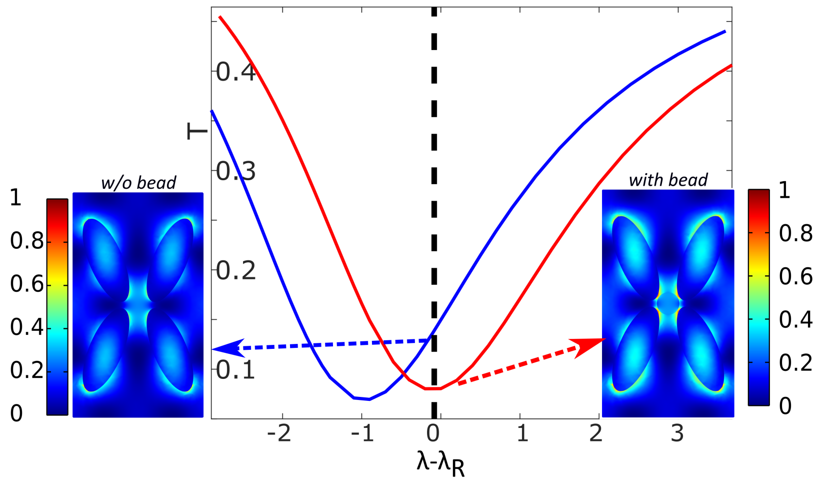

Figure S10: Transmission spectra of the unloaded nanostructure (blue) and the loaded nanostructure (red), showing the spectral red-shift associated with SIBA trapping.

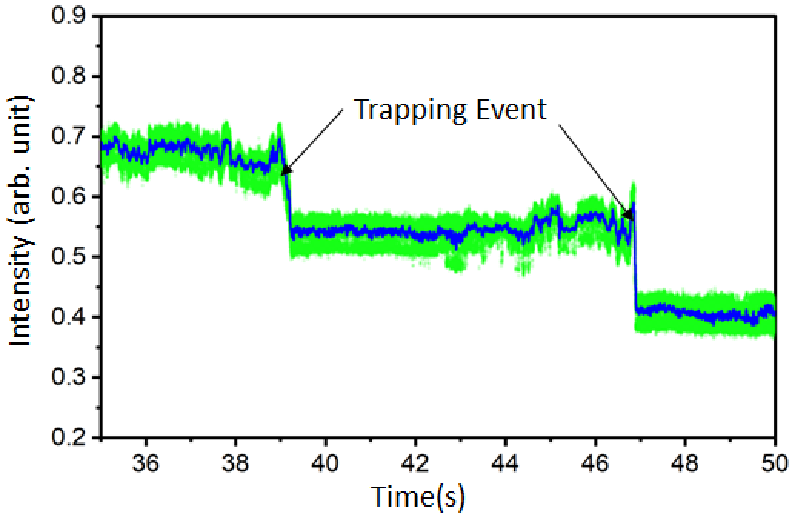

Figure S11: Step change indicating SIBA in action for two trapping events. The superimposed blue curve represents a moving average of data points.

S11: SIBA effect in the quadrupole array

We simulated the resonance spectrum of the nanostructure under two scenarios: one in the absence of nanoparticles and another in their presence. As shown in Fig. S10, the transmission spectrum of the loaded nanostructure shows a red-shift from the unloaded spectrum, which is characteristic of self-induced back action (SIBA). The presence of a particle within the quadrupole gap locally alters the effective index of the unit cell, resulting

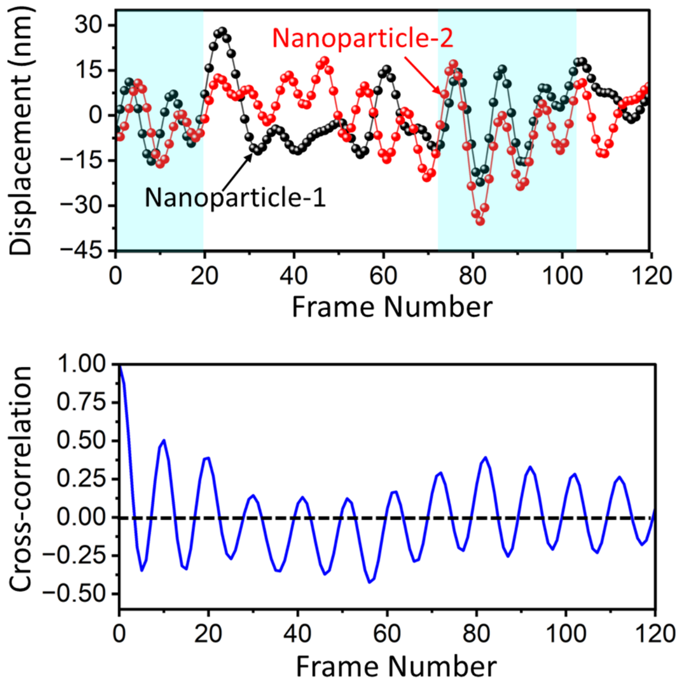

Figure S12: Experimentally determined dynamics for particles' movement (a) trajectories of centroid position of two particles trapped at adjacent sites, and (b) cross-correlation function.

in an approximate 1 nm redshift in the resonance position. To maximize the SIBA effect, we assumed the laser wavelength matched the resonance position of a complete trap (λ_R). It is worth noting that even when the trap is vacant, the array remains close to the resonance condition, resulting in only a two-fold reduction in energy confinement. This property proves advantageous in attracting particles initially moving around the traps.

This observation aligns qualitatively with the expected spectral red-shift due to the SIBA effect.

We have performed an initial analysis of the correlated motion of multiple trapped particles. In Fig. S12, we show the trajectories of two particles simultaneously trapped on different sites of the quadrupole array. As can be seen in shaded regions, the two particles' motion shows some correlation over limited timescales, further indicating the presence of nanostructure-mediated interactions between the particles. This will be the focus of future work.

Movies

Movie S1. Fano resonance assisted near field trapping of a single 100 nm polystyrene nanoparticles.

Movie S2. Simultaneous trapping of two individual 100 nm polystyrene nanoparticles at the adjacent (diagonal) sites.

References

(1) Yesilkoy, F.; Arvelo, E. R.; Jahani, Y.; Liu, M.; Tittl, A.; Cevher, V.; Kivshar, Y.; Altug, H. Ultrasensitive hyperspectral imaging and biodetection enabled by dielectric metasurfaces. *Nat. Photonics* **2019**, *13*, 390–396.

(2) Liu, M.; Choi, D.-Y. Extreme Huygens' metasurfaces based on quasi-bound states in the continuum. *Nano Lett.* **2018**, *18*, 8062–8069.

(3) Xu, Z.; Song, W.; Crozier, K. B. Optical trapping of nanoparticles using all-silicon nanoantennas. *ACS Photonics* **2018**, *5*, 4993–5001.

(4) Kotsifaki, D. G.; Chormaic, S. N. Plasmonic optical tweezers based on nanostructures: fundamentals, advances and prospects. *Nanophotonics* **2019**, *8*, 1227–1245.

(5) Wang, K.; Schonbrun, E.; Steinvurzel, P.; Crozier, K. B. Trapping and rotating nanoparticles using a plasmonic nano-tweezer with an integrated heat sink. *Nat. Commun.* **2011**, *2*, 469.
